# Prediction of Chronic Fracture Pain in Patients with Osteoporotic Fractures Using the Japanese Short-form Central Sensitization Inventory: A Single-center Retrospective Observational Study in a Convalescent Rehabilitation Ward

**DOI:** 10.1298/ptr.E10312

**Published:** 2025-01-29

**Authors:** Shotaro TAMURA, Sayo MIURA, Ryo MATSUDA

**Affiliations:** 1 Department of Rehabilitation, IMS Group IMS Sapporo Internal Medicine Rehabilitation Hospital, Japan; 2 Department of Rehabilitation, Japan Health Care College, Japan; 3 Department of Physical Therapy, Faculty of Health Sciences, Hokkaido University of Science, Japan

**Keywords:** Chronic fracture pain, Osteoporotic fractures, Short form Central Sensitization Inventory, Convalescent rehabilitation ward, Chronic pain assessment

## Abstract

Objectives: Chronic fracture pain (CFP) is a significant issue in patients with osteoporotic fractures (OFs) in convalescent rehabilitation wards (CRWs). This study aimed to examine the association between CFP and the Japanese short-form Central Sensitization Inventory (CSI-9) and verify the predictive validity of CSI-9 in patients with OF admitted to a CRW. Methods: This single-center retrospective study included 71 patients with OF (median age: 85.3 years, 54 females). CFP was defined as pain of Numerical Rating Scale (NRS) score ≥4 persisting at discharge, despite >3 months post-fracture. Multiple logistic regression and receiver operating characteristic curve analyses were performed to assess the association and predictive validity of the CSI-9 for CFP. Results: The incidence of CFP was 38.0%. CSI-9 was independently associated with CFP at admission (odds ratio = 1.12, 95% confidence interval [CI]: 1.01–1.24) and discharge (odds ratio = 1.15, 95% CI: 1.03–1.29). The area under the curve for the CSI-9 was 0.727 (95% CI: 0.605–0.850) at admission and 0.752 (95% CI: 0.637–0.867) at discharge, indicating fair predictive accuracy. The optimal cutoff values for the CSI-9 were 8 points at admission and 6 points at discharge. Conclusions: CSI-9 was independently associated with CFP and demonstrated moderate predictive accuracy in patients with OF in CRWs. Assessing central sensitization-related symptoms using the CSI-9 may be useful for evaluating and preventing CFP in this population. Further validation using large-scale prospective studies is required.

## Introduction

Japan is experiencing rapid population aging^1)^. Consequently, the incidence of osteoporotic fractures (OFs), such as vertebral compression and hip fractures, is increasing^2,3)^. OFs are considered a significant healthcare challenge.

OFs are the leading cause of long-term care needs^4)^. OF reduces an individual’s capacity for activities of daily living (ADL) and increases the risk of long-term care^5)^. Recently, the number of patients with musculoskeletal disorders and the proportion of older adults in convalescent rehabilitation wards (CRWs) has increased^6)^. Therefore, it is crucial to consider more appropriate assessment and intervention methods for elderly patients with OF in CRWs.

Pain associated with fractures is often chronic and is referred to as chronic fracture pain (CFP)^7)^. CFP is reportedly associated with physical function, ADL^8–11)^, depression, and quality of life (QOL). The incidence of chronic pain is particularly high in patients with vertebral compression and proximal femoral fractures. According to a study by Venmans et al.^10)^, 40% of patients with conservatively treated vertebral compression fractures still experience moderate or severe pain after 1 year. In a study of hip fracture patients, Salpakoski et al.^12)^ found that 42% of community-dwelling older adults (aged 60–85 years) experienced severe lower body pain 0.7–7.5 years post-fracture, with 70% of these individuals reporting severe pain in the operated hip. CRWs typically cater to patients with conditions such as stroke, orthopedic diseases, and disuse syndrome, allowing for hospitalizations of up to 180 days. In Japan, these wards have unique characteristics—they provide a maximum of 9 units of rehabilitation per day (with each unit lasting 20 minutes), enabling OF patients to receive intensive rehabilitation for approximately 3 months after onset. This comprehensive rehabilitation system aims to improve patients’ functional recovery and facilitate their return home. Given this extended rehabilitation period, assessing and managing chronic pain becomes particularly important for patients in CRWs. However, no study has focused on chronic pain in patients with OF, specifically in those in CRWs.

In 2022, chronic pain classification codes were added to the International Classification of Diseases, 11th Revision (ICD-11)^13)^. This has led to the international recognition of chronic pain as a disease. Central sensitization is an underlying mechanism of chronic pain. It is defined as “an amplification of neural signaling within the central nervous system that elicits pain hypersensitivity”^14)^. Although central sensitization is considered to be related to chronic pain, the clinical relationship between CFP and central sensitization has not been fully elucidated. The Central Sensitization Inventory (CSI) is a widely used screening tool for central sensitization-related symptoms^15)^. CSI is associated with pain intensity^16–19)^ and QOL^16,19)^ in chronic pain. The Japanese short-form Central Sensitization Inventory (CSI-9) has been developed^20)^ and is widely used for chronic pain treatment in Japan. However, the relationship between CSI and CFP in CRWs has not been examined.

Despite the high incidence of chronic pain after OF, the associated factors and predictive indicators remain unclear. Identifying predictive indicators for CFP is important for preventing the transition to chronic pain and avoiding long-term deterioration of patient outcomes. This study aimed to elucidate the relationship between CFP and CSI-9 and its predictive validity in patients with OF in CRWs.

## Methods

### Study design and participants

This was a single-center retrospective cohort study. The subjects were 192 patients aged 75 years or older with OF admitted to the CRWs of IMS Sapporo Internal Medicine Rehabilitation Hospital between April 2021 and March 2024, who required more than 3 months from injury to discharge from the CRWs.

We defined OF in this study as including both hip and vertebral compression fractures, the main orthopedic conditions in CRWs^21)^. This inclusive approach captures a broader representation of OFs in older adults, allowing for a larger sample size. While these fractures differ, they share common risk factors and consequences in the elderly. To address potential differences, we included fracture type as a covariate in our multivariate analysis, as separate subgroup analyses were challenging due to sample size limitations.

Excluded patients were those with a Mini-Mental State Examination (MMSE) score below 21, patients whose condition worsened during hospitalization, making follow-up difficult, and patients with missing data. The MMSE cutoff score of 21 was chosen as it is a commonly used threshold for mild cognitive impairment. While the validity of CSI-9 has not been specifically examined across different MMSE scores, we referred to studies on other self-report measures used in older adult populations. For instance, Allgaier et al.^20)^ found that the Geriatric Depression Scale (GDS) showed good validity in nursing home residents with MMSE scores of 15 or above. Although CSI-9 is not specifically designed for older adults like the GDS, this finding suggests that individuals with mild to moderate cognitive impairment may still provide valid responses on self-report measures.

### Ethical considerations

This study was conducted in accordance with the Declaration of Helsinki and was approved by the Ethics Committee of IMS Sapporo Internal Medicine Rehabilitation Hospital (approval number: 100). Due to the retrospective nature of the study, the requirement for individual informed consent was waived. Instead, an opt-out approach was adopted. Information about the study was publicly posted on the official website, and patients were allowed to opt out of the study. This opt-out method was approved by the ethics committee as part of the study protocol.

All data were anonymized before analysis to protect patient privacy. The study adhered to the ethical guidelines for medical and health research involving human subjects set by the Ministry of Health, Labour and Welfare of Japan.

### Survey items

The following items, collected and evaluated by physical therapists, were extracted from medical records:

Basic information included age, sex, body mass index (BMI), diagnosis, C-reactive protein (CRP), fracture classification (hip fracture or vertebral compression fracture), surgical procedure or not, MMSE score, days from injury to hospitalization in the CRW, days from injury to discharge from the CRW, and days from admission to CRW to discharge.

ADLs were evaluated using the motor items, cognitive items, and total score of the Functional Independence Measure (FIM) at admission and discharge.

The Short Physical Performance Battery (SPPB) was extracted as a measure of physical function. The SPPB consists of three subtests: balance test, 4-meter walking speed, and 5-time chair stand test. Each subtest is scored from 0 to 4 points, with a total score ranging from 0 to 12 points. Higher scores indicate better physical function^21)^.

Pain was assessed at admission and discharge using the Numerical Rating Scale (NRS) for pain intensity, the CSI-9 for central sensitization-related symptoms, and the Japanese short version of the Pain Catastrophizing Scale (PCS-6) for catastrophic thinking. The presence or absence of CFP at discharge was also evaluated.

The NRS ranged from 0 (no pain at all) to 10 (worst imaginable pain), and patients were asked to rate “the most severe pain experienced recently.”

The CSI-9, developed by Nishigami et al.^22)^, is a 9-item questionnaire with a total score of 36. Each item is rated on a 5-point scale: 0 (never), 1 (rarely), 2 (sometimes), 3 (often), and 4 (always). Higher scores indicate notable central sensitization-related symptoms. This scale has sufficient internal consistency and reliability.

The PCS-6, also developed by Nishigami et al.^23)^, is a 6-item questionnaire with a total score of 24. Each item is rated on a 5-point scale: 0 (not at all), 1 (slight), 2 (moderate), 3 (great), and 4 (all the time). Higher scores indicate greater catastrophic thinking. This scale has sufficient internal consistency and reliability.

### Definition of CFP

In this study, CFP was defined as “a state in which pain of NRS 4 or higher persisted at discharge, despite more than 3 months having passed since the fracture.” This definition is based on the ICD-11 classification of chronic pain^13)^ and reports by Zhao et al.^7)^ and Steingrímsdóttir et al.^24)^

### Statistical analysis

Descriptive statistics were used to confirm the characteristics of all patients in the CFP and non-CFP groups. Categorical variables are shown as numbers (%) and continuous variables as medians (interquartile ranges). For comparisons between the CFP and non-CFP groups, Fisher’s exact test was used for categorical variables and the Mann–Whitney U test for continuous variables.

To clarify the relationship between CFP and CSI-9, a multiple logistic regression analysis (forced entry method) was performed with the presence or absence of CFP (dummy variable: CFP = 1, non-CFP = 0) as the objective variable and CSI-9 as the explanatory variable. The effects of the CSI-9 at admission and discharge were analyzed separately. To avoid multicollinearity, Model 1 used the CSI-9 score at admission as the explanatory variable, whereas Model 2 used the CSI-9 score at discharge.

To adjust for confounding factors and consider the small sample size due to the frequency of CFP occurrence, we applied the propensity score covariate adjustment method by inputting the propensity score as an explanatory variable. The validity of the propensity score analysis method was determined based on the report by Austin^25)^. This method is effective in reducing the influence of confounding factors in observational studies, particularly in cases with small sample sizes or rare outcomes. In this study, we adopted the method of adjusting the propensity score as a covariate. This method controls for confounding similar to other propensity score methods and has the advantage of being directly usable in regression models. Considering our study’s sample size and number of variables, we determined this method to be appropriate. The propensity score was calculated using multiple logistic regression analysis with the presence or absence of CFP as the objective variable and age, sex (dummy variable: male = 1, female = 0), BMI, CRP, fracture site (dummy variable: proximal femoral fracture = 1, vertebral compression fracture = 0), surgical procedure or not, MMSE score, days from injury to hospitalization, FIM motor items at admission, SPPB total score, and NRS at admission as explanatory variables.

For the sensitivity analysis, a modified Poisson regression analysis was performed with the presence or absence of CFP as the objective variable and CSI-9 and propensity score as explanatory variables to calculate the risk ratio.

The predictive validity of the CSI-9 at admission and discharge for the presence or absence of CFP at discharge was analyzed using receiver operating characteristic (ROC) curves. To compare the performance of CSI-9 with other evaluation indicators in predicting CFP, ROC curves for PCS-6 were also generated. The cutoff value was determined based on the Youden Index. The Youden index is calculated by subtracting 1 from the sum of sensitivity and specificity, and it indicates the point that optimizes the balance between sensitivity and specificity. Using this cutoff value, we may be able to efficiently identify patients at high risk for CFP. The area under the curve (AUC) was calculated as an indicator of predictive accuracy, with 0.5 ≤ AUC < 0.6 judged as Fail, 0.6 ≤ AUC < 0.7 Poor, 0.7 ≤ AUC < 0.8 Fair, 0.8 ≤ AUC < 0.9 Good, and 0.9 ≤ AUC Excellent^26)^.

Statistical analysis was performed using R4.4.0 (Freeware, CRAN, Vienna, Austria) with a significance level of 5%.

### Sample size

The sample size was calculated based on the event per variable (EPV) rule proposed by Peduzzi et al.^27)^ to ensure the stability and reliability of the logistic regression model. In this study, because we performed multiple logistic regression analysis using two explanatory variables, the minimum number of events required was 20 (two variables × 10 events). Assuming a 40% CFP incidence rate based on previous studies, the minimum sample size was calculated. Therefore, a minimum of 50 participants was required for this study.

## Results

**Fig. 1.**
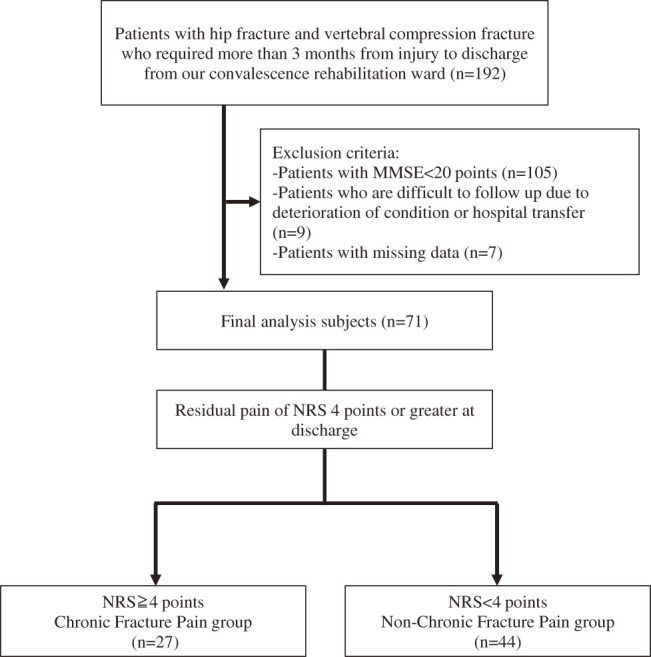
Flow diagram of subject selection MMSE, Mini-Mental State Examination; NRS, Numerical Rating Scale

A flowchart of the participant selection process is shown in Figure 1. The analysis included 71 patients (median age: 85.3 years [interquartile range: 81.0–89.0 years], 54 females). In all, 27 patients (38.0%) had CFP at discharge. As shown in Table 1, there were no significant differences in baseline characteristics, fracture site, or physical function and activity assessments between the CFP and non-CFP groups. However, pain assessment showed significantly higher NRS and CSI-9 scores in the CFP group at both admission and discharge.

**Table 1.** Group comparisons of baseline characteristics and clinical outcomes of participants

	Total	CFP group	Non-CFP group	p Value
n (%)	71	27	44	
Baseline at admission				
Age (years)	85.0 [81.0, 89.0]	87.0 [81.5, 89.5]	85.0 [81.0, 89.0]	0.585
Sex				0.252
Female	54 (76.1)	23 (85.2)	31 (70.5)	
Male	17 (23.9)	4 (14.8)	13 (29.5)	
Fracture classification				0.221
Vertebral compression fracture	37 (52.1)	17 (63.0)	20 (45.5)	
Hip fracture	34 (47.9)	10 (37.0)	24 (54.5)	
Surgical procedure or not				0.327
Surgical procedure	38 (53.5)	26 (59.1)	12 (44.4)	
Not	33 (46.5)	18 (40.9)	15 (55.6)	
Body mass index	21.1 [19.1, 23.6]	21.8 [19.7, 23.9]	20.9 [18.7, 23.4]	0.276
C-reactive protein (mg/dl)	0.39 [0.22, 0.81]	0.43 [0.15, 0.83]	0.37 [0.26, 0.80]	0.991
Mini-Mental State Examination (points)	25.0 [23.5, 28.0]	25.0 [23.5, 28.5]	25.0 [23.8, 27.0]	0.664
Days from injury to hospitalization (days)	23.0 [14.0, 36.0]	22.0 [12.0, 33.5]	23.5 [14.0, 37.3]	0.473
Functional Independence Measure (points)				
Total	66.0 [54.5, 75.5]	67.0 [54.5, 75.5]	63.5 [54.8, 73.8]	0.868
Motor items	35.0 [30.0, 44.0]	40.0 [33.0, 44.0]	33.5 [29.8, 43.3]	0.278
Cognitive items	30.0 [25.0, 35.0]	27.0 [23.0, 33.0]	31.0 [25.0, 35.0]	0.095
Short Physical Performance Battery (points)				
Walking speed	1.0 [1.0, 2.0]	1.0 [0.5, 2.0]	2.0 [1.0, 3.0]	0.304
5-time chair stand	0.0 [0.0, 0.5]	0.0 [0.0, 0.5]	0.0 [0.0, 0.3]	0.994
Standing balance	1.00 [0.0, 2.0]	1.0 [0.0, 2.5]	1.0 [0.0, 2.0]	0.628
Total	3.00 [1.0, 5.0]	3.0 [1.0, 5.0]	3.0 [1.0, 5.0]	0.867
Numerical Rating Scale (points)	5.0 [3.0, 7.0]	6.0 [5.0, 8.5]	5.0 [2.0, 7.0]	0.026
CSI-9 (points)	8.0 [4.0, 12.5]	11.0 [7.5, 14.0]	5.0 [3.0, 10.0]	p <0.001
PCS-6 (points)	9.0 [2.5, 14.0]	12.0 [4.0, 15.0]	6.5 [1.75, 13.0]	p <0.001
Clinical outcomes at discharge				
Hospitalization days (days)				
From injury to discharge from CRW	105.0 [96.0, 121.5]	105.0 [96.5, 116.0]	105.0 [95.8, 124.8]	0.713
From admission to CRW to discharge	86.0 [79.0, 90.0]	88.0 [83.5, 90.0]	86.0 [78.8, 90.0]	0.388
Functional Independence Measure (points)				
Total	110.0 [96.5, 116.5]	108.0 [90.0, 114.0]	112.0 [101.8, 118.0]	0.099
Motor items	78.0 [70.0, 83.5]	75.0 [61.5, 83.0]	79.0 [72.8, 85.0]	0.140
Cognitive items	33.0 [27.0, 35.0]	32.0 [26.0, 34.0]	33.5 [28.0, 35.0]	0.169
Short Physical Performance Battery (points)				
Walking speed	3.00 [2.0, 4.0]	2.0 [1.0, 4.0]	3.0 [2.0, 4.0]	0.354
5-time chair stand	1.00 [0.0, 3.0]	1.0 [0.0, 2.5]	1.0 [0.0, 3.0]	0.554
Standing balance	2.0 [2.0, 4.0]	2.0 [1.0, 4.0]	2.0 [2.0, 4.0]	0.545
Total	6.00 [4.0, 9.0]	7.0 [2.0, 9.0]	6.0 [5.0, 9.0]	0.385
Numerical Rating Scale (points)	3.0 [0.0, 5.0]	5.0 [5.0, 7.0]	0.0 [0.0, 2.0]	p <0.001
CSI-9 (points)	6.0 [3.0, 11.0]	9.0 [6.0, 13.5]	4.0 [1.0, 7.3]	p <0.001
PCS-6 (points)	4 [0.0, 9.5]	9.0 [3.5, 15.5]	2.0 [0.0, 5.3]	p <0.001

Numeric variable: Median [interquartile range], categorical variable: n (%).

CFP, chronic fracture pain; CSI-9, the Japanese short-form Central Sensitization Inventory; PCS-6, the Japanese short version of the Pain Catastrophizing Scale; CRW, convalescent rehabilitation ward

The results of multiple logistic regression analysis showed that the odds ratio in Model 1 was 1.12 (95% confidence interval [CI]: 1.01–1.24), indicating a significant independent association with CFP. Model 2 also showed a significant association with CFP, with an odds ratio of 1.15 (95% CI: 1.03–1.29) (Table 2). The c-statistic of the propensity score entered as a covariate was 0.720 (95% CI, 0.603–0.843), and the goodness of fit was within an acceptable range.

**Table 2.** Association between CFP and Central Sensitization Inventory: multiple logistic regression analysis

	Odds ratio	95% confidence interval	p Value
Model 1				
Intercept	0.04	0.01–0.21	p <0.001
CSI-9 at admission	1.12	1.02–1.25	0.023
Propensity score	97.3	5.8–2708.20	0.003
Model 2				
Intercept	0.05	0.01–0.23	p <0.001
CSI-9 at discharge	1.16	1.03–1.29	0.010
Propensity score	45.34	2.612–1151.25	0.013

Model 1: CSI-9 at admission as the independent variable; Model 2: CSI-9 at discharge as the independent variable.

Propensity Score: age, gender, body mass index, C-reactive protein, Mini-Mental State Examination, fracture classification, days from injury to hospitalization, motor and cognitive items on the functional independence measure at admission, Numerical Rating Scale at admission.

CFP, chronic fracture pain; CSI-9, the Japanese short-form Central Sensitization Inventory

The results of the modified Poisson regression analysis performed as a sensitivity analysis are presented in Table 3. The risk ratio in Model 1 was 1.053 (95% CI: 1.00–1.12), and that in Model 2 was 1.054 (95% CI: 1.00–1.11), showing a significant independent association with CFP (p <0.05), similar to the multiple logistic regression analysis.

**Table 3.** Association between CFP and Central Sensitization Inventory: modified Poisson regression analysis

	Relative risk ratio	95% confidence interval	p Value
Model 1				
Intercept	0.10	0.03–0.26	p <0.001
CSI-9 at admission	1.05	1.00–1.12	0.035
Propensity score	7.61	1.37–41.49	p <0.001
Model 2				
Intercept	0.11	0.04–0.28	p <0.001
CSI-9 at discharge	1.05	1.00–1.11	p <0.001
Propensity score	5.56	0.91–33.31	0.001

Model 1: CSI-9 at admission as the independent variable; Model 2: CSI-9 at discharge as the independent variable.

Propensity score: age, gender, body mass index, C-reactive protein, Mini-Mental State Examination, fracture classification, days from injury to hospitalization, motor and cognitive items on the functional independence measure at admission, and Numerical Rating Scale at admission.

CFP, chronic fracture pain; CSI-9, the Japanese short-form Central Sensitization Inventory

ROC curve analysis of the CSI-9 for CFP occurrence showed that the cutoff value based on the Youden Index was 8 points at admission and 6 points at discharge. The AUC was 0.727 (95% CI: 0.605–0.850) for CSI-9 at admission and 0.752 (95% CI: 0.637–0.867) for CSI-9 at discharge, both of which were considered fair. Sensitivity and specificity were similar at admission and discharge, with sensitivities of 70.4% and 70.5%, respectively. The AUC for PCS-6 was 0.620 (95% CI, 0.484–0.755) at admission, which was considered poor, and 0.745 (95% CI, 0.626–0.863) at discharge, which was considered fair (Table 4).

**Table 4.** Receiver operating characteristic curve analysis of CSI-9 and PCS-6

	CSI-9		PCS-6	
	Admission	Discharge	Admission	Discharge
The area under the curve	0.727	0.752	0.620	0.745
95% confidence interval	0.605–0.850	0.637–0.867	0.484–0.755	0.626–0.863
Cutoff value	8	6	11	5
Sensitivity	70.4%	70.4%	51.9%	66.7%
Specificity	70.5%	70.5%	70.5%	75.0%

CSI-9, the Japanese short-form Central Sensitization Inventory; PCS-6, the Japanese short version of the Pain Catastrophizing Scale

## Discussion

In this study, CSI-9 demonstrated a significant independent association with CFP and moderate predictive accuracy in patients with OF admitted to CRWs. This is the first study to examine early prediction of CFP using CSI-9 in the rehabilitation setting, targeting patients in the subacute-to-recovery phase rather than those with chronic conditions. These results suggest the utility of assessing central sensitization-related symptoms for evaluating and preventing CFP in this population.

The incidence of CFP in this study (38.0%) is similar to those reported in previous long-term follow-up studies^10,12)^. Despite the relatively early median time since onset (105 [interquartile range = 96.0–121.5] days), this high incidence of CFP is noteworthy. This result suggests that CFP occurs at a high rate, even in the intensive rehabilitation environment of CRWs, emphasizing the importance of CFP prevention during the recovery period.

The median age of the participants was 85 years (interquartile range = 81.0–89.0), with a large proportion of very old individuals. Advanced age is considered a risk factor for chronic pain^28,29)^, and the proportion of very old individuals in CRWs is expected to increase further with the aging population. Therefore, the importance of preventive interventions for CFP in patients in CRWs is likely to increase.

The significant association between CSI-9 and CFP highlights the importance of considering central sensitization-related symptoms when assessing CFP levels in CRW. Previous studies^30,31)^ have also reported an association between CSI and chronic pain; however, these studies targeted chronic-stage patients. A novel finding of the present study is the similar trend observed in patients admitted to the CRW relatively early after injury. The modified Poisson regression analysis showed a trend similar to that of the multiple logistic regression analysis. This finding confirms the robustness of the study’s main results. Modified Poisson regression analysis is useful for directly estimating risk ratios rather than odds ratios, especially when the incidence of an event is high^32)^. Considering the incidence of CFP in this study, the results of the sensitivity analysis provide important complementary information.

Regarding the mechanism linking CSI-9 and CFP, central sensitization may contribute to the maintenance and exacerbation of chronic pain. Central sensitization causes hyperalgesia and allodynia^33)^, which may lead to chronic pain after a fracture. Central sensitization is also closely related to psychosocial factors^34,35)^, which may have a combined effect on the occurrence of CFP. Patients with chronic pain and central sensitization are known to have reduced responsiveness to local treatments^36)^, which may influence the choice of rehabilitation approach during the recovery period. In recent years, the effectiveness of pain neuroscience education^37)^ and behavioral medicine approaches^38,39)^ as interventions for chronic pain has been reported, and these approaches may also be effective for patients with chronic pain with central sensitization.

The cutoff values for the CSI-9 in this study were 8 points at admission and 6 points at discharge. These values differ from the cutoff value (10 points) for discriminating central sensitization in a previous study^22)^. This discrepancy may be because this study aimed to predict CFP, while the previous study aimed to discriminate the presence or absence of central sensitization.

The results of the ROC curve analysis showed that the AUCs of the CSI-9 were 0.727 at admission and 0.752 at discharge, both of which were considered fair, suggesting the existence of limitations in predicting CFP using the CSI-9 alone. However, no previous study has longitudinally examined the usefulness of the CSI-9 in predicting the chronicity of pain in patients with OF. The findings of our study provide useful information for planning CFP prevention and treatment in CRWs. To compare the performance of the CSI-9 with other assessment measures in predicting CFP, an ROC curve analysis of PCS-6 was performed. The results showed that the predictive accuracy of the PCS-6 at admission was poor (AUC, 0.620; 95% CI = 0.484–0.755) and was significantly lower than that of the CSI-9. This result emphasizes the important role of CSI-9 in predicting CFP. The difference in predictive capability between CSI-9 and PCS-6 can be interpreted as reflecting the distinct nature of the constructs measured by these two scales. Pain catastrophizing, as assessed by PCS-6, tends to fluctuate with pain experiences. At admission, many patients are experiencing acute severe pain, which may lead to generally higher PCS-6 scores, potentially reducing its discriminative power in predicting CFP. On the other hand, CSI-9 evaluates symptoms related to central nervous system sensitization, which is thought to more directly reflect the mechanisms of pain chronification. Therefore, CSI-9 is presumed to have demonstrated a more stable predictive ability compared to PCS-6. These differences in the underlying constructs measured by each scale likely account for the observed disparity in their CFP prediction capabilities.

Central sensitization plays a crucial role in the development and maintenance of chronic pain. From the perspective of neuroplasticity, persistent nociceptive stimuli increase the excitability of neurons in the dorsal horn of the spinal cord, leading to hyperalgesia and allodynia^40)^. Additionally, dysfunction of the descending pain inhibitory system is involved in central sensitization, with reports of altered endogenous opioid systems in chronic pain patients^41)^. These complex mechanisms may also be relevant in the context of CFP, potentially contributing to the development of chronic pain following fractures.

Screening with CSI-9 may enable early identification of chronic pain risk, potentially allowing for the consideration of individualized intervention approaches. For instance, patients with high central sensitization risk might benefit from strategies such as enhanced pain neuroscience education^42)^, cognitive-behavioral therapy approaches^43)^, or aerobic exercises aimed at exercise-induced hypoalgesia^44)^. However, as this is an observational study, caution should be exercised when inferring causal relationships.

### Limitations

This study had several methodological limitations. First, this was a single-center retrospective cohort study and caution should be exercised regarding its generalizability. Facility-specific treatment protocols and patient characteristics may have influenced the results, and future multicenter studies are required to validate the study findings. Second, although propensity scores were used to adjust for confounding factors, unmeasured confounders may have been present. Potential confounding factors, such as comorbidities, pharmacotherapy, and other psychosocial factors, may have influenced CFP occurrence, and future studies should involve comprehensive analyses that consider these factors. Third, this study did not examine the impact of CFP on patients’ long-term functional recovery and QOL. Previous studies have reported that chronic pain in older adults is associated with limitations in ADL, reduced social participation, sleep disturbances, and exacerbation of depressive symptoms^8–11)^. Chronic pain may lead to decreased physical activity and increased risk of falls, particularly in the elderly, potentially accelerating the frailty cycle^45)^. Future longitudinal studies are needed to track the trajectories of long-term functional recovery and changes in QOL based on the presence or absence of CFP. Fourth, this study lacks detailed records on specific intervention techniques. However, all patients received a standardized amount of rehabilitation (approximately 9 units daily) in the same CRW environment. While we cannot fully account for the impact of intervention variability, the consistency in rehabilitation quantity and setting likely minimized its effect on outcomes. Future studies should document intervention details to enable more comprehensive analysis. Fifth, comprehensive psychosocial assessment is absent beyond PCS-6. While PCS-6 measures pain catastrophizing, it does not evaluate other crucial factors such as depression or anxiety. This limitation restricts our understanding of the full spectrum of psychosocial influences on CFP development. Future studies should incorporate a broader range of psychosocial measures to conduct more comprehensive analyses. Finally, it was difficult to infer causality in this study. Future prospective cohort studies that consider additional potential confounders and intervention studies examining the effectiveness of CFP prevention interventions involving the CSI-9 in CRWs are warranted.

## Conclusion

This study is the first to attempt to predict CFP using the CSI-9 in patients with OF admitted to CRWs. Although many previous studies have targeted patients with chronic-stage CFP, this study targeted patients with subacute-to-recovery-stage CFP, demonstrating the novelty of early prediction and preventive interventions for CFP.

The results showed that the incidence of CFP was 38.0%. Furthermore, CSI-9 was independently associated with CFP regardless of age, fracture site, and pain at admission and had moderate predictive accuracy. These results suggest the usefulness of the CSI-9 for pain assessment in CRW.

In the future, it will be necessary to validate the findings of this study through large-scale, multicenter, prospective studies. Furthermore, we aimed to verify the effectiveness of preventive interventions based on screening using the CSI-9. These efforts are expected to lead to the development of new strategies for preventing and managing CFP in patients with OF admitted to a CRW.

## Acknowledgments

We would like to express our sincere gratitude to the patients and staff of IMS Sapporo Internal Medicine Rehabilitation Hospital for their cooperation in this study. We especially thank the physical therapists in the rehabilitation department for their dedicated efforts in data collection. We are also deeply grateful to Mr. Ryo Matsuda, Assistant Professor at Hokkaido University of Science, for his invaluable guidance and supervision throughout this research project. We would like to thank Editage (www.editage.jp) for editing the English language.

## Funding

Not applicable.

## Conflicts of Interest

The authors declare no potential conflicts of interest with respect to the research, authorship, and publication of this article.
